# An Investigation of Helicopter Parenting and Interpersonal Conflict in a Competitive College Climate

**DOI:** 10.3390/healthcare11101484

**Published:** 2023-05-19

**Authors:** Ting Nie, Mingyang Cai, Yan Chen

**Affiliations:** School of Business, Macau University of Science and Technology, Macau 999078, China; tnie@must.edu.mo (T.N.); 3220002990@student.must.edu.mo (M.C.)

**Keywords:** helicopter parenting, psychological entitlement, fear of missing out, interpersonal conflict, competitive climate

## Abstract

With declining birth rates, and decreasing family sizes being witnessed around the world, helicopter parenting—characterized by high warmth, high control, and low empowerment—is becoming increasingly common among parents. Parenting styles exert long-term effects on individuals’ cognition and behavior, not only in early childhood, but also in adulthood: therefore, within this context, this study explored the underlying influence mechanism of helicopter parenting style on interpersonal conflict, through a survey of 505 Chinese college students. Using multiwave data, our analysis uncovered the mediating roles of psychological entitlement and fear of missing out, as well as the moderating role of a competitive climate. In particular, we found that helicopter parenting enhances interpersonal conflict among college students, by strengthening psychological entitlement and fear of missing out. In addition, the indirect effect of fear of missing out is stronger than that of psychological entitlement. We also found that a competitive climate positively moderates the indirect effect of helicopter parenting on interpersonal conflict among college students through psychological entitlement and fear of missing out, indicating that the negative effects of helicopter parenting are more pronounced in a high competitive climate. These results provide a novel theoretical account of how early parenting styles affect an adult’s cognition and behavior. Practically, these results suggest that parents should limit the use of helicopter parenting, and that, while loving and caring for their child, they should allow their child appropriate autonomy. On the other hand, children should strive for positive self-improvement and harmonious peer relationships, to alleviate the negative influence of helicopter parenting.

## 1. Introduction

Children’s cognition and personalities are shaped by their parents’ parenting styles. Moreover, this influence extends from early childhood into adulthood [1]. As an emerging parenting style, helicopter parenting has received a lot of attention from researchers recently. Helicopter parenting refers to an overprotective and highly involved parenting style, in which parents provide unlimited love and support to their child, but also display overprotective behaviors, and therefore deny their child the autonomy the child deserves [2]. Due, in part, to the implementation of the one-child policy, this style of parenting is becoming increasingly prevalent in China. The so-called one-child policy, which was in effect in China from 1978 to 2016, restricted many families to only having one child. Under this policy, it has been observed that an only child usually becomes the center of his or her family, and receives care and attention from all family members, including parents and grandparents. As a result, these children are typically raised under the full protection of their parents, receiving substantial spiritual and financial support. The advantage is that the parents of these families are able to devote a great deal of time, energy, and financial resources to raising their child, to ensure for their child the most conducive environment in which to grow up and achieve success in the future [3]. On the other hand, these families are faced with new challenges. Many parents experience high levels of anxiety, which may negatively impact their child’s development. Because of such anxiety, parents hover over their child like helicopters, ready to make decisions and take responsibility for them [4]. With many countries around the world facing declining birth rates and smaller family sizes, helicopter parenting is becoming increasingly common. It is generally agreed by researchers that helicopter parenting is an inappropriate parenting style that is practiced by parents to ensure their child’s success and well-being [2], but which has negative effects on the parent–child relationship, and on the child’s personality, mental health, and academic performance [5,6]. However, the influence mechanism underlying helicopter parenting’s intrinsic effects has received little attention in the existing literature, and therefore remains unclear. Motivated by this research gap, this study aimed to investigate the effect of helicopter parenting on interpersonal conflicts among college students.

Higher education is a transitional stage between youth and adulthood. Even though most individuals are learning to be independent at this stage, the influence of their families remains significant. The effects of parenting can still be seen, occasionally, in their pursuit of academic excellence and their development of social networks. For example, the excessive care and protection displayed by parents in helicopter parenting may inadvertently result in their child having distorted self-perceptions. Consequently, these children are more likely to develop a sense of entitlement [7], and an inflated belief in their own eligibility for favorable outcomes, compared to that of their peers [8]. Additionally, they typically want to repay their parents’ high expectations with commendable academic performance [9]. As these children transition to independent living in college, those with a stronger sense of self are more likely to believe they are worthy of higher recognition and rewards [10]. Due to this psychological entitlement, individuals may attribute positive and successful events to themselves, and negative events and failures to others or to external factors, such as their environment [11]. Moreover, after entering college, these individuals must face academic pressure and interpersonal relationships autonomously; without the comprehensive arrangements and protection of their parents that they are used to, their need for guidance in their actions is not fully satisfied, and they are prone to feeling as if they are missing out [12]. Such individuals tend to seek more resources and information, to relieve the anxiety and insecurity stemming from their separation from their parents; aside from which, as they grow up and become independent, their attachment to their parents gradually shifts towards attachment to their peers [13]. Thereby, such individuals, accustomed to parental protection, tend to search for new peer attachments more actively at this stage. Once they have formed these connections, they also tend to exhibit a high level of dependence on their peers. They are eager to gain more support and recognition, and are concerned about being isolated and negatively evaluated in their peer group [14], which generates a strong fear of missing out. This results in them spending more time and energy on others than on themselves. When their peers have more experience or knowledge than they do, they are likely to become more concerned. Because of this, they experience higher levels of anxiety and stress when interacting with others [15], which is detrimental to maintaining harmonious interpersonal relations.

In recent years, college students have faced a more serious situation of involution than ever before, while pursuing their studies and careers. Involution is described as people competing for limited resources, and entering an internal conflict, very much like “vicious competition”, which in turn results in declining “effort-to-reward ratios” for individuals [16]. This irrational and excessive competitive behavior or phenomenon is prevalent in higher education institutions, where the availability of quality educational resources is typically limited. In college, students are constantly competing with their peers for educational resources and development opportunities, such as guaranteed graduate school admission, opportunities to study abroad, and scholarship quotas. Drawing on social comparison theory, competition is essentially a pressure to compare oneself to others [17]. Involution intensifies college competition and mutual comparisons among students, which can be detrimental to students who are overprotected by their parents. Those affected by helicopter parenting may be more adversely affected by involution if they are not sufficiently prepared, either cognitively or competently.

Helicopter parenting has existed for several generations in China, and it is prevalent around the globe as well: in spite of this, a clear understanding about its influence mechanisms is still lacking. This study investigated the influence mechanisms of the helicopter parenting style on individual interpersonal conflict, through a survey of 505 Chinese college students. Our results reveal the mediating effect of psychological entitlement and fear of missing out, as well as the moderating effect of a competitive climate.

## 2. Literature Review and Hypotheses

Helicopter parenting is characterized by high warmth/support, high control, and low autonomy-giving parenting behaviors [4]. Children who are subject to this parenting style can clearly feel the love of their parents, who are supportive in various ways, including emotionally, monetarily, and by being giving of their time [18]. On the other hand, these parents also display overprotective behaviors, and fail to give their child age-appropriate autonomy [2]. Control is a key feature of helicopter parenting. Unlike authoritarian parenting, helicopter parents monitor their child’s behavior, to ensure that the child can develop according to the parents’ expectations, and they avoid scenarios they consider to be risky, as their child grows up [19]. Helicopter parents rarely demonstrate psychological control over their child, and do not rely on emotions, such as manipulation, guilt, and withdrawal, for control purposes: instead, they more often demonstrate behaviors intended to shape their child for their child’s own good [2]. Helicopter parenting is often the result of parental projections and expectations. More specifically, helicopter parents hope that their child will achieve the parents’ own unrealized ideals, and therefore give conditional attention to their child, by over-controlling their child’s life, in order to compensate for their own regrets [20]. In addition, helicopter parenting is often driven by parental separation anxiety, in which parents exhibit both caring and controlling characteristics, due to the fear of separation from their child [21]. Some studies have examined the effects of helicopter parenting on the cognition and behavior of youth and emerging adults. Reed et al. [22] found that helicopter parenting is often associated with low levels of self-efficacy. Children raised by helicopter parenting are prone to questioning their own abilities, which is unfavorable to the development of a positive self-concept. Carone et al. [23] reported that helicopter parenting negatively affects the child’s well-being, as it infringes on the child’s need for autonomy. Some other studies have pointed out that children raised by helicopter parenting are prone to higher levels of anxiety, and tend to use inappropriate coping styles. Therefore, the helicopter parenting style is significantly predictive of both internet addiction and alcohol addiction behaviors [24].

Interpersonal conflict refers to disagreement between individuals or groups [25]. When individuals interact with others, if their goals and expectations are interfered with by the actions of others, negative emotional feelings arise: this leads to behaviors that disrupt harmonious interpersonal relationships, and interpersonal conflict is thus generated [26]. Barki and Hartwick [27] pointed out that interpersonal gaps have three attributes—disagreement, negative emotion, and interference—which reflect different elements of interpersonal conflict, in terms of cognition, emotion, and behavior, respectively. Based on these three attributes, interpersonal conflict can further be defined as a dynamic process that occurs between two interdependent parties: in this dynamic process, both parties experience negative emotions when they perceive differences of opinion or obstacles to the accomplishment of the goal. Interpersonal conflict is prevalent among both adolescents and employees: it is strongly associated with a range of negative outcomes for individuals and teams [28]. Frequent interpersonal conflict is a threat to individual mental health and well-being [29], which is especially true for adolescents with strong peer attachments. Adolescents’ perception of interpersonal conflict can contribute to a large number of negative behaviors, and even to suicidal intentions [30]. Interpersonal conflict increases dissonance within teams, decreases cooperation and communication among team members, and adversely affects knowledge sharing and employee creativity [28,31]. Existing empirical studies [27] have shown that hostile environments, difficult tasks, and certain personal traits, such as neuroticism and perfectionism, are critical factors contributing to interpersonal conflict. In college, the influence of parents and family parenting on students remains significant. Helicopter-parenting parents are overly involved in their child’s life, interfering with the child’s problem solving, and limiting the child’s autonomy. Numerous studies have found a significant positive association between helicopter parenting and children’s depression and anxiety [32], which prevents harmonious relationships with peers, and increases interpersonal conflict. Based on the discussion above, we developed Hypothesis 1 as follows:

**Hypothesis** **1.** *Helicopter parenting positively affects interpersonal conflict among college students.*

‘Psychological entitlement’ refers to an individual’s belief or perception that said individual deserves more social resources and preferential treatment than others, an expectation that is not realistic, and which lacks rationality [33]. There is some debate as to whether individual psychological entitlement is stable. Campbell [33] identified it as a stable personality trait that is contextually consistent and temporally stable, and does not change according to situations, or over time. Zitek et al. [34] argued that psychological entitlement reflects a state of being, which is influenced by a variety of factors, and exhibits unstable characteristics. An individual’s psychological entitlement is closely related to his or her childhood experiences, social status, and parental relationships. Childhood misfortune tends to spawn self-compassion. Parental disharmony, single-parent family, disabilities, and not being treated with respect or equality can lead individuals to believe that they deserve preferential treatment [10,35]. Such individuals may even feel that they deserve more resources than others, whether they try or not [34]. Individuals with higher social class and economic status are prone to higher feelings of superiority, and to narcissistic tendencies, and thus tend to have higher psychological entitlement [36,37]. These personality trait tendencies are harmful to individual development, due to self-centeredness and lack of empathy, and often lead to maladaptive behaviors, such as aggressive behavior, unethical behavior, and exploitative behavior [38,39].

Helicopter parenting entails high support and low autonomy. In helicopter parenting, parents provide unconditional support to their child, and are prone to coddling, which may enhance their child’s psychological entitlement [40]. In addition, children subject to this parenting style tend to be more externally controlled: they believe that external factors, such as luck, chance, and power, are responsible for the outcome of things [11]. Furthermore, individuals who are externally controlled have higher levels of psychological entitlement than individuals who exercise internal control [41]. Moreover, individuals with high psychological entitlement desire more resources and preferential treatment, tend to attribute negative outcomes to others, and are more likely to blame and alienate their peers; their interpersonal distress is more severe, because of their unwillingness to show tolerance or take the initiative to mend relationships [42]. Moeller et al. [43] also demonstrated that psychological entitlement has a significant positive relationship with perceived interpersonal conflict and perceived hostility. Therefore, we proposed Hypothesis 2:

**Hypothesis** **2.** *Psychological entitlement has a mediating role in the relationship between helicopter parenting and interpersonal conflict among college students.*

‘Fear of missing out’ describes the anxiety that individuals feel when they perceive that their peers are having more positive experiences, or possess more information than they do [15]: as Przybylski et al. [44] define it, this is a diffuse anxiety, caused by fear of missing out on important experiences of others. As a result, individuals spend more time paying high levels of attention to other people’s dynamics. Fear of missing out reflects an individual’s fear that others will have rewarding experiences that they do not have, as well as a desire to stay in regular contact with the people in that individual’s social network. Psychologically, this fear of missing out is a desire for interpersonal relationships. Anxiety arises as a result of unmet interpersonal longings, when the individual misses out on meaningful experiences or real-time dynamics with others [45]. Everyone is born with varying degrees of tendency towards fear of missing out. The level experienced by each individual is influenced by certain factors, such as their environment and interpersonal relationships [46]. Extraversion and agreeableness positively predict fear of missing out, while neuroticism attenuates individuals’ fear of missing out [47]. Individuals with a high need for belonging and popularity tend to be more concerned about their social connections and interpersonal situations; therefore, they are more likely to experience fear of missing out. The phenomenon of the fear of missing out is also explained as an unstable state of self-regulation, caused by a lack of psychological need satisfaction [48,49]. As individuals with fear of missing out are always anxious about missing out on novel experiences or favorable opportunities, it is considered to be a source of negative emotions, such as depression. According to Barry and Wong [50], high fear of missing out is associated with negative self-perceptions, loneliness, low self-esteem, and low self-compassion.

Helicopter parenting is characterized by the parents having a high level of control over their child’s behavior: as a result, the child’s need for autonomy is suppressed. Failing to meet an individual’s psychological needs will inevitably result in impaired self-regulation and unbalanced cognitive development, ultimately leading to a feeling of missing out [51]. This perceived anxiety, arising from fear of missing out, is an important factor inducing perceived stress [48,52], and is not conducive to harmonious interpersonal relationships. Specifically, individuals with a high fear of missing out are worried about falling behind others, which makes them very concerned about what other people are doing, or what the most recent trends are. The emergence of fear of missing out may exacerbate an individual’s problematic use of social networks. Individuals may spend too much time online, focusing on others’ information, and may alleviate negative emotions at the cost of exacerbated offline interpersonal stress and conflict [53]. Based on the discussion above, we proposed Hypothesis 3, as follows.

**Hypothesis** **3.** *Fear of missing out has a mediating role on the relationship between helicopter parenting and interpersonal conflict among college students.*

Competition is a universal phenomenon in today’s society. The concern about a competitive climate originated in business management. Brown et al. [17] defined a competitive climate as an organizational climate in which employees feel competitive, and feel pressured to compare their pay or rewards to those of their colleagues. In general, it can be observed in the comparison of employee performance to that of colleagues, in the perception of other employees’ competitive status, or in the way that different employees respond to incentives differently. Thus, a competitive climate is essentially pressure to compare oneself to others. The perception of such a competitive climate leads to a higher degree of competition pressure [54]. In the literature, there has been controversy regarding the role of such competitive environments. Some scholars argue that the perception of a competitive atmosphere has positive effects on employees. In particular, a competitive environment encourages employees to make efforts to express themselves. Moreover, employees working in a competitive environment may be intrinsically motivated by the act of comparing themselves to their colleagues. A competitive environment may encourage employees to be more self-motivated to deal with challenges at work, resulting in higher levels of work engagement and performance [55,56]. Some other scholars argue that a competitive climate in organizations can sometimes be destructive, leading to negative work behaviors and lower job engagement. As the nature of competition is a struggle for scarce resources, the victory of one party is based on the defeat of his or her competitors. Therefore, in a highly competitive climate, individuals are more likely to engage in self-serving behavior, to realize their own benefits [57]. In addition, high levels of competition further exacerbate employees’ doubts about their self-worth, and their concerns about their status, which leads to more performance- and goal-oriented actions, and aggravates unethical behavior in the workplace [58].

Similarly, in learning environments, such as colleges, high levels of competition may enhance the individual’s focus on outcomes, and lead to self-affirmation and self-transcendence through comparison of self to others [17]. Helicopter parents tend to overprotect their child, thereby reducing their ability to cope with high levels of competition. Children with helicopter parents usually believe that they deserve preferential treatment and more benefits than others [10]. Individuals with this high psychological entitlement are more likely to feel dissatisfied with rules, outcomes, and rewards: consequently, they have more negative experiences, and more conflicts with others in competitive environments [43]. A competitive environment further reduces the possibility of individual privileges in the allocation of limited resources in colleges, thus leading to more conflicts. On this basis, we proposed Hypotheses 4 and 5:

**Hypothesis** **4.** *A competitive college climate has a moderating role between psychological entitlement and interpersonal conflict among college students.*

**Hypothesis** **5.** *A competitive college climate positively moderates the indirect effect of helicopter parenting on interpersonal conflict among college students through psychological entitlement.*

Under helicopter parenting, parents exert high control over their child, and their child’s autonomy is thereby reduced [2]. In addition, when these basic psychological needs are not met, individuals will become more concerned with the status of their peers [51]. An intense competitive climate further exacerbates this high degree of social comparison. As a result, the adverse effects of role pressure are amplified under such circumstances [59]. Individuals will tend to spend more time and energy tracking others’ information, and making constant comparisons: this, in turn, leads to higher anxiety, which is not conducive to maintaining healthy interpersonal relationships. In sum, a competitive climate has a more adverse impact on individuals who grow up in an overprotective and controlling environment, and thus have lower traits of competitiveness [60]. On this basis, we proposed Hypotheses 6 and 7, as follows.

**Hypothesis** **6.** *A competitive college climate has a moderating role between fear of missing out and interpersonal conflict among college students.*

**Hypothesis** **7.** *A competitive college climate positively moderates the indirect effect of helicopter parenting on interpersonal conflict among college students, through fear of missing out.*

## 3. Method

### 3.1. Participants and Sampling Procedure

To verify the effect of helicopter parenting on interpersonal conflict among college students, a survey was used, to examine the hypothesized dual mediating influence mechanism. The survey was conducted online through Wenjuanxing.com, a major online survey platform in China. The participants for this survey were limited to college students, and most of them were from the Guangdong, Guangxi, Hunan, Hubei, Yunnan, and Guizhou provinces of China. During the survey, the participants were informed of the study’s aim, and were assured that the data collected would only be used for academic research purposes. The questionnaire was completed anonymously by the participants, who were free to terminate the survey at any time. Incomplete questionnaires were considered invalid, and were excluded from further analysis. In addition, to reduce the effect of potential common method bias, two rounds of data collection were conducted, with an interval of one month between them. In the first round of data collection (T1), the questionnaires were collected through convenience sampling, and were used to measure the helicopter parenting, interpersonal conflict, competitive climate, and demographic information provided by the respondents. The total number of questionnaires distributed was 800, of which, 694 valid questionnaires were recovered, and the effective recovery rate was 86.7%. In the second round of data collection (T2), the questionnaires were distributed to the same group of participants, to measure their psychological entitlement and their fear of missing out. During Round 2, a total of 694 questionnaires were distributed. After data cleaning and response matching, 505 valid questionnaires were recovered. The effective recovery rate of the questionnaires was 72.8% in Round 2.

The demographic characteristics of the respondents in this study were as follows. In terms of gender, males accounted for 49.1% (248) of respondents, females accounted for 50.9% (257) of respondents, and the gender ratio was relatively balanced; in terms of age, the age distribution of the respondents ranged from 17 to 25 years old, with an average age of 20.73. All of the respondents were college or university students. Students enrolled in vocational college accounted for 25.5% (129) of the respondents; students enrolled in university undergraduate programs accounted for 63.2% (319) of the respondents; and students enrolled in university graduate programs accounted for 11.3% (57) of the respondents. Of the total number of respondents, 66.9% (338) were from the natural sciences majors, and 33.1% (167) were from the humanities and social sciences majors. Most of the respondents (76.8% (388)) grew up in one-child families.

### 3.2. Measures

Self-report scales were used in this study, which employed Likert 5-point scales, from “complete disagreement” to “complete agreement”.

#### 3.2.1. Helicopter Parenting

Helicopter parenting emphasizes high warmth/support, high control, and low autonomy-giving parenting behaviors [4]. Helicopter parenting was measured using a 5-item scale, developed by Padilla-Walker and Nelson [21], e.g., “My parents will help me with any crisis or problem I may encounter”: in this study, the Cronbach’s alpha coefficient of this scale was 0.720.

#### 3.2.2. Psychological Entitlement

‘Psychological entitlement’ describes an individual’s unrealistic and irrational belief or perception that said individual deserves more social resources and preferential treatment than do others [33]. Psychological entitlement was measured using a 4-item scale developed by Campbell [33], e.g., “I deserve more from life”: in this study, the Cronbach’s alpha coefficient of this scale was 0.858.

#### 3.2.3. Fear of Missing Out

‘Fear of missing out’ describes the anxiety that individuals feel due to the fear that their peers will have more positive experiences or have more information than they do [15]. Fear of missing out was measured using a 10-item scale developed by Scott & Bruce, e.g., “I’m afraid that my friends have more experience than I do”: in this study, the Cronbach’s alpha coefficient of this scale was 0.851.

#### 3.2.4. Interpersonal Conflict

‘Interpersonal conflict’ describes disagreement between individuals or between groups [25]. The measurement of interpersonal conflict was adapted from a 5-item scale in the study of Spector and Jex [61], e.g., “I often have arguments with my classmates in college”: in this study, the Cronbach’s alpha coefficient of this scale was 0.751.

#### 3.2.5. Competitive Climate

‘Competitive climate’ refers to the climate of an organization in which employees feel competitive and pressured to compare their pay rewards to those of their colleagues [17]. The measurement of competitive climate was adapted from a 5-item scale used in the study of Fletcher et al. [60], e.g., “In college, My classmates often compare their performance with mine”: in this study, the Cronbach’s alpha coefficient of this scale was 0.841.

#### 3.2.6. Control Variables

Gender, education, major, and family type were considered as control variables. The classifications were as follows: gender (male; female); education (vocational program; undergraduate program; graduate program); major (natural sciences, humanities, and social sciences); family type (one-child families and non-one-child families).

## 4. Results

### 4.1. Confirmatory Factor Analysis

In the confirmatory factor analysis (CFA), AMOS 24 was used to test the instrument validity. The result showed that the overall model fit of the five-factor model (helicopter parenting, psychological entitlement, fear of missing out, interpersonal conflict, and competitive climate) was acceptable ($\chi^{2}/df$ = 1.795, GFI = 0.926, CFI = 0.956, IFI = 0.957, RMSEA = 0.040, and RMR = 0.055): this indicated good discriminant validity among the main variables. The model fit of the single-factor model was far from acceptable ($\chi^{2}/df$ = 6.635, GFI = 0.693, CFI = 0.678, IFI = 0.681, RMSEA = 0.106, and RMR = 0.098). In addition, the common method deviation in the study was not a significant concern.

### 4.2. Descriptive Statistics and Correlation Analysis

SPSS 26.0 was used for descriptive statistics and correlation analysis. The results are shown in Table 1. When controlling the effects of gender, education, major, and family type, helicopter parenting was significantly associated with psychological entitlement (r = 0.343, *p* < 0.01), fear of missing out (r = 0.484, *p* < 0.01), and interpersonal conflict (r = 0.474, *p* < 0.01). In addition, psychological entitlement was significantly associated with interpersonal conflict (r = 0.467, *p* < 0.01); fear of missing out was significantly associated with interpersonal conflict (r = 0.577, *p* < 0.01); and competitive climate was significantly associated with helicopter parenting (r = 0.316, *p* < 0.01), psychological entitlement (r = 0.246, *p* < 0.01), fear of missing out (r = 0.320, *p* < 0.01), and interpersonal conflict (r = 0.233, *p* < 0.01). Preliminary correlation analysis results support our research hypotheses.

### 4.3. Hypothesis Testing

Hayes’ PROCESS macro 3.6 (Model 4) was used to test the main effect and mediating effects between helicopter parenting, psychological entitlement, fear of missing out, and interpersonal conflict. Bootstrapping with 5000 repetitions was performed, to determine 95% confidence intervals of the direct and indirect effects. The results are summarized in Table 2. First of all, the results show that helicopter parenting was significantly associated with interpersonal conflict (0.317, 0.052), and that the 95% confidence interval of this direct effect was [0.216, 0.419], which excluded zero. This result indicates that helicopter parenting positively affects interpersonal conflict among college students, and that Hypothesis 1 is supported. Secondly, the 95% confidence interval of the mediating effect (0.074, 0.026) of psychological entitlement between helicopter parenting and interpersonal conflict was [0.027, 0.128]. This confidence interval determined by bootstrapping also excluded zero, suggesting that helicopter parenting affects interpersonal conflict among college students through psychological entitlement. Therefore, Hypothesis 3 is supported. Thirdly, the 95% confidence interval of the mediating effect (0.227, 0.037) of fear of missing out, between helicopter parenting and interpersonal conflict, was [0.157, 0.303]. Similarly, the confidence interval determined by bootstrapping excluded zero, indicating that helicopter parenting affects interpersonal conflict among college students through fear of missing out. Thus, Hypothesis 3 is further supported. Moreover, the 95% confidence interval of the indirect effect contrast (−0.152, 0053), between psychological entitlement and fear of missing out, was [−0.258, −0.050], which suggests that the indirect mediating effect of fear of missing out, between helicopter parenting and interpersonal conflict, is significantly stronger than that of psychological entitlement.

As a next step, Hayes’ PROCESS macro 3.6 (Model 14) was used, to test the moderating effects and moderated mediating effects of competitive climate. Table 3 presents the testing results. Firstly, the 95% confidence interval of the interaction between competitive climate and psychological entitlement (0.146, 0.055) was [0.038, 0.255], which excluded zero: this suggests that a competitive climate has a moderating effect on the relationship between psychological entitlement and interpersonal conflict. At the low level of competitive climate, the direct effect of psychological entitlement on interpersonal conflict was significant (0.278, 0.056), with a 95% confidence interval [0.169, 0.388], which excluded zero. At the high level of competitive climate, the direct effect of psychological entitlement on interpersonal conflict was significant (0.454, 0.053), with a 95% confidence interval [0.350, 0.558] excluding zero as well. These results indicate that higher competitive climates strengthen the influence of psychological entitlement on interpersonal conflict among college students. In addition, the 95% confidence interval of the moderated mediating effect (0.060, 0.033) of competitive climate among helicopter parenting, psychological entitlement, and interpersonal conflict was [0.010, 0.139] (excluding zero), which suggests that competitive climate positively moderates the indirect effect of helicopter parenting on interpersonal conflict among college students, through psychological entitlement. At the low level of competitive climate, the indirect mediating effect of psychological entitlement between helicopter parenting and interpersonal conflict was significant (0.113, 0.032), with a 95% confidence interval [0.049, 0.174] (excluding zero). At the high level of competitive climate, the indirect mediating effect of psychological entitlement, between helicopter parenting and interpersonal conflict, was significant (0.185, 0.036), with a 95% confidence interval [0.123, 0.256] (excluding zero). Altogether, these results demonstrate that a higher competitive climate strengthens the indirect effect of helicopter parenting on interpersonal conflict among college students, through psychological entitlement. Accordingly, Hypotheses 4 and 5 are supported.

Secondly, the 95% confidence interval for the interaction between competitive climate and fear of missing out (0.174, 0.056) was [0.064, 0.284] (excluding zero), which suggests that competitive climate has a moderating effect on the relationship between fear of missing out and interpersonal conflict. At the low level of competitive climate, the direct effect of fear of missing out on interpersonal conflict was significant (0.365, 0.056), with a 95% confidence interval [0.255, 0.476] (excluding zero). At the high level of competitive climate, the direct effect of fear of missing out on interpersonal conflict was significant (0.575, 0.053), with a 95% confidence interval [0.470, 0.679] (excluding zero). These results demonstrate that a higher competitive climate strengthens the influence of fear of missing out on interpersonal conflict among college students. Furthermore, the 95% confidence interval of the moderated mediating effect of competitive climate (0.104, 0.043) among helicopter parenting, fear of missing out, and interpersonal conflict, was [0.032, 0.199]: this 95% confidence interval excluded zero, which suggests that a competitive college climate positively moderates the indirect effect of helicopter parenting on interpersonal conflict among college students, through fear of missing out. At the low level of competitive climate, the indirect mediating effect of fear of missing out, between helicopter parenting and interpersonal conflict, was significant (0.218, 0.045), with a 95% confidence interval [0.127, 0.305] (excluding zero). At the high level of competitive climate, the indirect mediating effect of fear of missing out, between helicopter parenting and interpersonal conflict, was significant (0.343, 0.044), with a 95% confidence interval [0.265, 0.438] (excluding zero). These results demonstrate that a higher competitive climate strengthens the indirect effect of helicopter parenting on interpersonal conflict among college students, through fear of missing out. Thus, Hypotheses 6 and 7 are supported.

## 5. Conclusions and Discussion

This study aimed to explore the influence mechanism of helicopter parenting on interpersonal conflict among college students, using the multiwave data of Chinese college students. Our results revealed the underlying double-mediating mechanism between helicopter parenting and interpersonal conflict among college students. Specifically, the mediating effects of both psychological entitlement and fear of missing out are existent in the relationship between helicopter parenting and interpersonal conflict among college students. In other words, helicopter parenting exacerbates interpersonal conflict among college students, by enhancing both psychological entitlement and fear of missing out, with the indirect effect of fear of missing out being stronger than that of psychological entitlement. In addition, a competitive college climate positively moderates the indirect effect of helicopter parenting on interpersonal conflict among college students (through psychological entitlement and fear of missing out), such that the negative effects of helicopter parenting are more pronounced in a high competitive climate.

Helicopter parenting is characterized by three characteristics: high warmth; high behavioral control; and low authority [6]. In line with most previous research, this study confirmed that helicopter parenting adversely affects children from an interpersonal perspective [62]. Some studies argue that helicopter parents provide their child with as much support and opportunity as they can, due to their love and high expectations [63], which may improve their child’s skills and competitiveness in the future. However, this study found that high parental warmth and support also result in negative effects, leading to the child’s failure to develop correct and rational self-perception. As a result of being overprotected by their parents, children tend to overestimate their own abilities and values. They also expect to be the focus of their group at all times. After they enter university and start to live in a group, they are more likely to have a psychological gap, when not able to secure attention and special treatment: if this occurs when they want to obtain a resource advantage, it may, in turn, intensify their conflict with their peers. Thus, helicopter-parenting-raised children may be unable to show strong competitiveness as they become independent. Rather, they may have too high an opinion of themselves to maintain harmonious relationships with others.

Moreover, it has been demonstrated by previous studies that high behavioral control and low authorization lead to inadequately met needs in individuals. It has also been discovered that, as children develop independence, they often adapt, to make up for any unfulfilled need, by managing their relationships with their peers [64]. Furthermore, children raised by helicopter parenting experience intense parental care and high expectations from an early age. As a result, they display a strong desire to succeed in many areas, such as academia, to gain parental appreciation and recognition. They strive not to do wrong things or to fall behind their peers, to avoid disappointing their parents [65]. Therefore, they will be especially concerned if others possess more valuable information and/or have more positive experiences than they do [45]. This study argues that this fear of missing out can create a very ambivalent psychology. Due to this ambivalent psychology, individuals are overly concerned about others, and tend to compare themselves with others. With this level of excessive concern and attention, individuals tend to dedicate most of their time and energy to gathering information and making comparisons, which leads to anxiety and ruminative thinking, as well as to addictive behaviors, such as excessive use of social media [66]. The resulting lack of interaction with peers or friends further impairs the long-term development of successful interpersonal relationships.

This study also found that the indirect effect of helicopter parenting on interpersonal conflict through fear of missing out is stronger than that of psychological entitlement. Our conclusion, that high control and low empowerment have more pronounced negative effects on children, is consistent with the findings on authoritarian parenting in previous studies [67]. Helicopter parenting differs from authoritarian parenting, in that the children of helicopter parents are able to feel the love of their parents, and psychological control is not exercised heavily [68]; however, due to their lack of autonomy, children raised by helicopter parenting may develop an over-dependence on their parents, which can negatively affect both their sense of control and their sense of security. The negative effects of this psychological deficit may persist, even after independence, in a variety of ways, including academic, interpersonal, and career-related.

Competition indicates that people are compared to each other, and that harmonious relationships are challenged [69]. Both negative and positive effects of competitive environments have been reported in the existing literature. This study found that a competitive climate can reinforce the negative effect of helicopter parenting on interpersonal conflict. Specifically, children growing up with overprotective and behaviorally controlling parents tend to have cognitive and behavioral inconsistency, i.e., feeling positive about themselves while being overly concerned about others. In a competitive environment, resources are limited; therefore, it is more difficult for individuals to maintain a dominant position or obtain special treatment in a group for a long time. These children may suffer psychologically from a lack of privilege in such a circumstance, resulting in more conflict between them. In addition, competition also makes each individual more concerned about the resource they have, and reinforces a self-protective mindset, which makes it more difficult to obtain information about others. As a result, individuals will undoubtedly feel more anxious, and will spend more time gathering information, which is also not conducive to maintaining healthy interpersonal relationships.

Over the past three years, the COVID-19 pandemic has greatly affected families across the globe. As a result of changes in work, life, and learning styles, parents and their children have experienced extraordinary pressure. Several studies have demonstrated that helicopter parenting in such extreme circumstances may have more adverse consequences on children’s mental health and conduct, potentially impairing their ability to develop the psychological resilience needed to cope with adversity [70,71,72]. In the foregoing context, this study found that helicopter parenting leads to significant negative consequences for college students. In particular, helicopter parenting leads to increased interpersonal conflict, which can be detrimental to children in an interdependent society; therefore, efforts should be made to reduce its negative effects, on both sides—parents and children. From a parent’s perspective, parents should avoid helicopter parenting as much as possible. In spite of the fact that parents are motivated by love for their child, and wish to prepare their child for a successful future, they do not have the right to deprive that child of the basic necessities of life. A child’s mental health depends on the satisfaction of many needs, such as physical needs, safety needs, social needs, respect needs, and self-actualization. Any suppressed or deprived needs may cause deficiencies in the child’s personality, thus affecting that child’s future development. Furthermore, despite being a continuation of their parents’ lives, children are independent individuals, who do not have to carry out their parents’ dreams. It should be made clear that parents should not transfer their anxieties to their child. Eventually, all children need to face their own pressures in life, independently. The best gift parents can give their child is to develop their child’s confidence, strength of will, and abilities to cope with future challenges, rather than being a helicopter parent, always hovering over them. From a child’s perspective, individuals should take the initiative to alleviate the negative influence of their original family. Children need to be grateful for their parents’ love and attention; however, they also need to be conscious of the fact that they are in charge of their own future. It is impossible for children to rely on their parents completely throughout their lives, so they must work hard to improve their skills and abilities, and increase their competitiveness. Moreover, as social beings, everyone should be able to build their own social network, and to benefit from it. The transition from parental attachment to peer attachment is an inevitable stage in human growth; therefore, for better self-development, individuals should strive to build harmonious and high-value social relationships.

## 6. Limitations and Future Study

There are some limitations to the findings of this study. Firstly, all of the samples studied were from China. Helicopter parenting is more common in one-child families, and the culture of paternal and child superiority makes this parenting style more acceptable in China. These factors render China different to many other countries, which may have caused some external validity issues for this study. Secondly, some of the scales used in this study (e.g., interpersonal conflict, competitive climate) are not specific to higher education institutions, due to a lack of widely accepted measurement instruments in the relevant field, for college students. Despite some similarities in interpersonal conflict and competitive climate across different scenarios, the accuracy and relevance of measurement can be somewhat affected. Finally, self-reported scales were used in the study. Although our analysis indicated that the common method bias is not of major concern, more accurate measures could be considered in future studies.

As an emerging parenting style, helicopter parenting has gained widespread attention in recent years. In particular, with birth rates declining and family sizes decreasing worldwide, helicopter parenting is likely to become more prevalent. Some studies have reported certain negative effects of helicopter parenting; however, to date, its underlying influence mechanisms remain unclear. Our results show that it not only exerts short-term effects on the cognition and behavior of adolescents, but also generates long-term effects on aspects such as work and marriage in adulthood. Future studies may consider follow-up interviews or use longitudinal data to gain a comprehensive understanding of the role that helicopter parenting plays throughout an individual’s life. In addition, the causes of helicopter parenting are worth exploring in depth. Personal traits, regional policies, social pressures, and family characteristics may all contribute to helicopter parenting, which needs to be verified in future studies.

## Figures and Tables

**Table 1:** Descriptive Statistics and Correlation Analysis (N = 505).

	Mean	SD	1	2	3	4	5	6	7	8	9
Gender	1.51	0.500	-								
Education	2.86	0.601	−0.009	-							
Major	1.33	0.471	−0.227 **	0.141 **	-						
family	1.77	0.422	0.212 **	−0.142 **	−0.103 *	-					
HP	3.06	0.702	−0.340 **	0.063	0.165 **	−0.206 **	**0.665**				
PE	3.36	0.833	−0.308 **	0.086	0.202 **	−0.212 **	0.343 **	**0.736**			
FMO	3.15	0.866	−0.414 **	0.096 *	0.215 **	−0.244 **	0.484 **	0.607 **	**0.741**		
IC	2.52	0.915	−0.488 **	0.017	0.181 **	−0.208 **	0.474 **	0.467 **	0.577 **	**0.703**	
CC	3.60	0.600	−0.260 **	0.098 *	0.103 *	−0.136 **	0.316 **	0.246 **	0.320 **	0.233 **	**0.678**

Note: ** *p* < 0.01, * *p* < 0.05; HP: Helicopter Parenting, PE: Psychological Entitlement, FMO: Fear of Missing Out, IC: Interpersonal Conflict; CC: Competitive Climate. The bold value on the diagonal is the AVE root value of each variable.

**Table 2:** Mediating Effect (N = 505).

	Path	Effect	SE	LLCI	ULCI
Direct Effect	HP→IC	0.317	0.052	0.216	0.419
Total Indirect Effect	HP→PE/FMO→IC	0.301	0.036	0.232	0.376
Indirect Effect	HP→PE→IC	0.074	0.026	0.027	0.128
Indirect Effect	HP→FMO→IC	0.227	0.037	0.157	0.303
Contrast	IE(PE)-IE(FMO)	−0.152	0.053	−0.258	−0.050

Note: HP: Helicopter Parenting; PE: Psychological Entitlement; FMO: Fear of Missing Out; IC: Interpersonal Conflict; IE: Indirect Effect; LLCI: Lower level of 95% confidence interval; ULCI: Upper level of 95% confidence interval.

**Table 3:** Moderating Effects and Moderated Mediating Effects (N = 505).

Variable		Effect	SE	*p*	LLCI	ULCI		Index	SE	LLCI	ULCI
	Int_1	0.146	0.055	0.008	0.038	0.255		0.060	0.033	0.010	0.139
PE	L	0.278	0.056	0.000	0.169	0.388	L	0.113	0.032	0.049	0.174
	H	0.454	0.053	0.000	0.350	0.558	H	0.185	0.036	0.123	0.265
			Moderating Effect					Moderated Mediating Effect			
		Effect	SE	*p*	LLCI	ULCI		index	SE	LLCI	ULCI
	Int_1	0.174	0.056	0.002	0.064	0.284		0.104	0.043	0.032	0.199
FMO	L	0.365	0.056	0.000	0.255	0.476	L	0.218	0.045	0.127	0.305
	H	0.575	0.053	0.000	0.470	0.679	H	0.343	0.044	0.265	0.438

Note: HP: Helicopter Parenting; PE: Psychological Entitlement; FMO: Fear of Missing Out; IC: Interpersonal Conflict; IE: Indirect Effect. LLCI: Lower level of 95% confidence interval, ULCI: Upper level of 95% confidence interval.

## Data Availability

The data presented in this study are available on request from the corresponding author.

## Abbreviations

The following abbreviations are used in this manuscript:

HP	Helicopter Parenting
PE	Psychological Entitlement
FMO	Fear of Missing Out
IC	Interpersonal Conflict
CC	Competitive Climate

## References

[1] Ge M., Sun X., Huang Z. (2022). Correlation between Parenting Style by Personality Traits and Mental Health of College Students. Occup. Ther. Int..

[2] Segrin C., Woszidlo A., Givertz M., Bauer A., Murphy M.T. (2012). The Association Between Overparenting, Parent-Child Communication, and Entitlement and Adaptive Traits in Adult Children. Fam. Relat..

[3] Carlsson F., Lampi E., Martinsson P., Tu Q., Yang X. (2022). Are only-children different? Evidence from a lab-in-the-field experiment of the Chinese one-child policy. PLoS ONE.

[4] LeMoyne T., Buchanan T. (2011). Does “Hovering” Matter? Helicopter Parenting and its Effect on Well-being. Sociol. Spectr..

[5] Schiffrin H.H., Liss M. (2017). The Effects of Helicopter Parenting on Academic Motivation. J. Child Fam. Stud..

[6] Shaki O., Gupta G.K., Yadav P., Faisal F.A. (2022). Helicopter parenting, from good intentions to poor outcomes. What parents needs to know?. J. Fam. Med. Prim. Care.

[7] Shadach E., Rappaport S., Dollberg D., Tolmacz R., Levy S. (2018). Relational entitlement, early recollections of parental care, and attachment orientation. Curr. Psychol..

[8] Candel O.S., Turliuc M.N. (2017). An overview of the research of psychological entitlement. Definitions and conceptual characteristics. Rev. Psihol..

[9] Benner A.D., Fernandez C.C., Hou Y., Gonzalez C.S. (2021). Parent and teacher educational expectations and adolescents’ academic performance: Mechanisms of influence. J. Community Psychol..

[10] Dreiling E.A. (2015). The Interrelationships among Perceived Parenting Styles, Psychological Entitlement, and Subjective Well-Being. Ph.D. Thesis.

[11] Kwon K.A., Yoo G., Bingham G.E. (2016). Helicopter Parenting in Emerging Adulthood: Support or Barrier for Korean College Students’ Psychological Adjustment?. J. Child Fam. Stud..

[12] Koca F., Saatçı F. (2022). The Mediator Role of Fear of Missing Out in the Parent-Adolescent Relationship Quality and Problematic Internet Use. Int. J. Ment. Health Addict..

[13] Fraley R.C., Davis K.E. (1997). Attachment formation and transfer in young adults’ close friendships and romantic relationships. Pers. Relatsh..

[14] Carr V.M., Francis A.P., Wieth M.B. (2021). The relationship between helicopter parenting and fear of negative evaluation in college students. J. Child Fam. Stud..

[15] Vaughn J., Mack A.M. (2012). Fear of Missing Out (FOMO).

[16] Edwards J., Ship A. (2007). The relationship between person-environment fit and outcomes: An integrative theoretical framework. Perspectives on Organizational Fit.

[17] Brown S.P., Cron W.L., Slocum J.W. (1998). Effects of trait competitiveness and perceived intraorganizational competition on salesperson goal setting and performance. J. Mark..

[18] Fitzgerald K.S. (2015). Helicopter parenting and parent-child attachment. Sr. Honor. Proj..

[19] Hong J.C., Hwang M.Y., Kuo Y.C., Hsu W.Y. (2015). Parental monitoring and helicopter parenting relevant to vocational student’s procrastination and self-regulated learning. Learn. Individ. Differ..

[20] Segrin C., Givertz M., Swaitkowski P., Montgomery N. (2015). Overparenting is Associated with Child Problems and a Critical Family Environment. J. Child Fam. Stud..

[21] Padilla-Walker L.M., Nelson L.J. (2012). Black hawk down?: Establishing helicopter parenting as a distinct construct from other forms of parental control during emerging adulthood. J. Adolesc..

[22] Reed K., Duncan J.M., Lucier-Greer M., Fixelle C., Ferraro A.J. (2016). Helicopter Parenting and Emerging Adult Self-Efficacy: Implications for Mental and Physical Health. J. Child Fam. Stud..

[23] Carone N., Gartrell N.K., Rothblum E.D., Koh A.S., Bos H.M. (2022). Helicopter parenting, emotional avoidant coping, mental health, and homophobic stigmatization among emerging adult offspring of lesbian parents. J. Fam. Psychol..

[24] Cui M., Allen J.W., Fincham F.D., May R.W., Love H. (2019). Helicopter Parenting, Self-regulatory Processes, and Alcohol Use among Female College Students. J. Adult Dev..

[25] Dahrendorf R. (1958). Toward a theory of social conflict. J. Confl. Resolut..

[26] Pinkley R.L., Northcraft G.B. (1994). Conflict frames of reference: Implications for dispute processes and outcomes. Acad. Manag. J..

[27] Barki H., Hartwick J. (2004). Conceptualizaing the Construct of Interpersonal Conflict. Int. J. Confl. Manag..

[28] Moldovan D., Bocos M. (2021). Resolving Interpersonal Conflicts through Collaboration-an Alternative Way to Facilitate the Social Integration of Students in Primary School. Rev. Rom. Pentru Educ. Multidimens..

[29] Darbonne A., Uchino B., Ong A. (2013). What Mediates Links Between Age and Well-being? A Test of Social Support and Interpersonal Conflict as Potential Interpersonal Pathways. J. Happiness Stud..

[30] Moyano N., Vélez K., Arias A., del Mar Sánchez-Fuentes M. (2022). Two pathways to suicidal intention in Ecuadorian adolescents: The role of parental and peer attachment, depression and impulsivity. Curr. Psychol..

[31] De Clercq D., Belausteguigoitia I. (2021). The links among interpersonal conflict, personal and contextual resources, and creative behaviour. Can. J. Adm. Sci. Rev. Can. Des Sci. L’Adm..

[32] Veronica D., Norvilitis J.M., Schuetze P. (2017). The Relationship between Helicopter Parenting and Adjustment to College. J. Child Fam. Stud..

[33] Campbell B., Campbell W.K., Bonacci A.M., Shelton J., Exline J.J., Bushman B.J. (2004). Psychological entitlement: Interpersonal consequences and validation of a self-report measure. J. Personal. Assess..

[34] Zitek E.M., Jordan A.H., Monin B., Leach F.R. (2010). Victim entitlement to behave selfishly. J. Personal. Soc. Psychol..

[35] Richardson E., Simons L., Futris T. (2017). Linking Family-of-Origin Experiences and Perpetration of Sexual Coercion: College Males’ Sense of Entitlement. J. Child Fam. Stud..

[36] Cai H., Kwan V., Sedikides C. (2012). A sociocultural approach to narcissism: The case of modern China. Eur. J. Personal..

[37] Matherne C.F., Credo K.R., Gresch E.B., Lanier P.A. (2019). Exploring the relationship between covert narcissism and amorality: The mediating influences of self-efficacy and psychological entitlement. Am. J. Manag..

[38] Eissa G., Lester S.W. (2022). A Moral Disengagement Investigation of How and When Supervisor Psychological Entitlement Instigates Abusive Supervision. J. Bus. Ethics.

[39] Qureshi A.R., Raza B. (2022). Impact of Psychological Entitlement and Prevention Work Regulatory Focus on Unethical Pro-Organizational Behaviors: Mediating Role of Reflective Moral Attentiveness. Abasyn Univ. J. Soc. Sci..

[40] Rothman A.M., Steil J.M. (2012). Adolescent attachment and entitlement in a world of wealth. J. Infant Child Adolesc. Psychother..

[41] Carnes A.M., Knotts K.G. (2018). Control and Expectancy: Locus of Control as a Predictor of Psychological Entitlement. Empl. Responsib. Rights J..

[42] Exline J.J., Baumeister R.F., Bushman B.J., Campbell W.K., Finkel E.J. (2004). Too Proud to Let Go: Narcissistic Entitlement as a Barrier to Forgiveness. J. Personal. Soc. Psychol..

[43] Moeller S.J., Crocker J., Bushman B.J. (2009). Creating hostility and conflict: Effects of entitlement and self-image goals. J. Exp. Soc. Psychol..

[44] Przybylski A.K., Murayama K., DeHaan C.R., Gladwell V. (2013). Motivational, emotional, and behavioral correlates of fear of missing out. Comput. Hum. Behav..

[45] Abel J.P., Buff C.L., Burr S.A. (2016). Social media and the fear of missing out: Scale development and assessment. J. Bus. Econ. Res. JBER.

[46] Wegmann E., Oberst U., Stodt B., Brand M. (2017). Online-specific fear of missing out and Internet-use expectancies contribute to symptoms of Internet-communication disorder. Addict. Behav. Rep..

[47] Arora D., Kaur M. (2019). Detach out to attach on everything: A study on relationship between personality and fear of missing out (FoMO). Indian J. Health Wellbeing.

[48] Beyens I., Frison E., Eggermont S. (2016). “I don’t want to miss a thing”: Adolescents’ fear of missing out and its relationship to adolescents’ social needs, Facebook use, and Facebook related stress. Comput. Hum. Behav..

[49] Alabri A. (2022). Fear of missing out (FOMO): The effects of the need to belong, perceived centrality, and fear of social exclusion. Hum. Behav. Emerg. Technol..

[50] Barry C.T., Wong M.Y. (2020). Fear of missing out (FoMO): A generational phenomenon or an individual difference?. J. Soc. Pers. Relatsh..

[51] Lai C., Altavilla D., Ronconi A., Aceto P. (2016). Fear of missing out (FOMO) is associated with activation of the right middle temporal gyrus during inclusion social cue. Comput. Hum. Behav..

[52] Chiou W.B., Lee C.C., Liao D.C. (2015). Facebook effects on social distress: Priming with online social networking thoughts can alter the perceived distress due to social exclusion. Comput. Hum. Behav..

[53] Fox J., Moreland J.J. (2015). The dark side of social networking sites: An exploration of the relational and psychological stressors associated with Facebook use and affordances. Comput. Hum. Behav..

[54] Locke E.A. (1968). Toward a theory of task motivation and incentives. Organ. Behav. Hum. Perform..

[55] David E.M., Kim T.Y., Rodgers M., Chen T. (2021). Helping while competing? The complex effects of competitive climates on the prosocial identity and performance relationship. J. Manag. Stud..

[56] Itani O.S., Chaker N.N. (2022). Harnessing the Power Within: The Consequences of Salesperson Moral Identity and the Moderating Role of Internal Competitive Climate. J. Bus. Ethics.

[57] Wisse B., Rus D., Keller A.C., Sleebos E. (2019). “Fear of losing power corrupts those who wield it”: The combined effects of leader fear of losing power and competitive climate on leader self-serving behavior. Eur. J. Work. Organ. Psychol..

[58] Irangani B.S., Liu Z., Kumar N., Khanal S. (2021). Effect of competitive psychological climate on unethical pro-team behavior: The role of perceived insider status and transformational leadership. Management.

[59] Buunk B.P., Van der Zee K., VanYperen N.W. (2001). Neuroticism and social comparison orientation as moderators of affective responses to social comparison at work. J. Personal..

[60] Fletcher T.D., Major D.A., Davis D.D. (2008). The interactive relationship of competitive climate and trait competitiveness with workplace attitudes, stress, and performance. J. Organ. Behav..

[61] Spector P., Jex S. (1998). Development of four self-report measures of job stressors and strain: Interpersonal Conflict at Work Scale, Organizational Constraints Scale, Quantitative Workload Inventory, and Physical Symptoms Inventory. J. Occup. Health Psychol..

[62] Jung E., Hwang W., Kim S., Sin H., Zhao Z., Zhang Y., Park J.H. (2020). Helicopter parenting, autonomy support, and student wellbeing in the United States and South Korea. J. Child Fam. Stud..

[63] Hesse C., Mikkelson A.C., Saracco S. (2018). Parent–child affection and helicopter parenting: Exploring the concept of excessive affection. West. J. Commun..

[64] Schiffrin H.H., Batte-Futrell M.L., Boigegrain N.M., Cao C.N., Whitesell E.R. (2021). Relationships between helicopter parenting, psychological needs satisfaction, and prosocial behaviors in emerging adults. J. Child Fam. Stud..

[65] Loughlin-Presnal J., Bierman K.L. (2017). How do parent expectations promote child academic achievement in early elementary school? A test of three mediators. Dev. Psychol..

[66] Servidio R. (2021). Self-control and problematic smartphone use among Italian University students: The mediating role of the fear of missing out and of smartphone use patterns. Curr. Psychol..

[67] Muhopilah P., Tentama F. (2021). The Model Influence of Authoritarian Parenting, Extraversion Personality, and Conformity to Bullying among Students. Int. J. Eval. Res. Educ..

[68] Odenweller K.G., Booth-Butterfield M., Weber K. (2014). Investigating helicopter parenting, family environments, and relational outcomes for millennials. Commun. Stud..

[69] Sofyan Y., De Clercq D., Shang Y. (2023). Detrimental effects of work overload on knowledge hiding in competitive organisational climates. Asia Pac. J. Hum. Resour..

[70] Munz E. (2022). Hovering over Graduate Students? The Relationship between Helicopter Parenting and Graduate Students’ Depression. Pa. Commun. Annu..

[71] Seki T., Haktanir A., Şimşir Gökalp Z. (2023). The mediating role of resilience in the relationship between helicopter parenting and several indicators of mental health among emerging adults. J. Community Psychol..

[72] Forresi B., Giani L., Scaini S., Nicolais G., Caputi M. (2023). The Mediation of Care and Overprotection between Parent-Adolescent Conflicts and Adolescents’ Psychological Difficulties during the COVID-19 Pandemic: Which Role for Fathers?. Int. J. Environ. Res. Public Health.

